# Exploring the Scientific Literature Between Ultraprocessed Foods and Cancer: A Scientometric Mapping

**DOI:** 10.1155/2024/3763197

**Published:** 2024-06-28

**Authors:** Fran Espinoza-Carhuancho, Carlos Quispe-Vicuña, Cesar Mauricio-Vilchez, Julia Medina, Luzmila Vilchez, Lucia Quispe-Tasayco, Frank Mayta-Tovalino

**Affiliations:** ^1^ Grupo de Bibliometría Evaluación de evidencia y Revisiones Sistemáticas (BEERS) Human Medicine Career Universidad Científica del Sur, Lima, Peru; ^2^ Academic Department Red de Eficacia Clínica y Sanitaria (REDECS), Lima, Peru; ^3^ Academic Department Faculty of Medical Technology Universidad Nacional Federico Villarreal, Lima, Peru; ^4^ Academic Department Research Innovation and Entrepreneurship Unit Universidad Nacional Federico Villarreal, Lima, Peru; ^5^ Vicerrectorado de Investigación Universidad San Ignacio de Loyola, Lima, Peru

## Abstract

**Objective:** The objective of the study is to explore the scientific literature between ultraprocessed foods (UPFs) and cancer using a scientometric mapping.

**Materials and Methods:** A Scopus search was conducted on February 4, 2024, limited to papers published between 2018 and 2023. We found 662 articles, 189 reviews, 68 book chapters, 13 conference papers, and 9 notes. The whole analysis included the evaluation of scholarly output by country/region, the number of scholarly papers produced (scholarly output), the number of views (view count), the field-weighted citation impact (FWCI).

**Results:** In the analysis conducted for the period 2018–2023, a dataset was examined where the annual growth rate was 5.96%, indicating a sustained expansion of the literature. The average number of citations per paper was 18.56, underlining the impact and relevance of the publications. Sixty-six single-authored papers were identified, and international collaborations accounted for 27.23% of the collaborative efforts. The most prominent authors were Inge Huybrechts, Marc J.R. Gunter, and Edward Luciano Giovannucci. In terms of impact and visibility, Harvard University leads with 52 contributions and a field-weighted impact of 3.39.

**Conclusions:** The literature in the field of UPFs and cancer has experienced a sustained expansion. The scientometric indicators reveal a high activity of recent academic contributions with significant impact.

## 1. Introduction

Cancer is one of the main diseases affecting the global population and in recent years has reported more than 19 million new cases, almost 10 million deaths due to cancer, and by 2040, it is expected to increase by almost 50% [1]. This high incidence and mortality of cancer is, in turn, conditioned by various risk factors such as smoking, alcoholism, and a high body mass index (BMI), which can increase cancer deaths by more than 20% [2]. In addition to these factors, diet should also be highlighted, since it has been reported that an increase in the diet of vegetables and fruits can reduce the risk of developing colorectal, breast, lung, and even prostate cancer [3, 4]. In this regard, ultraprocessed foods (UPFs) stand out as potential carcinogenic foods.

Globally, recent increases in both the availability and consumption of UPF have been reported, especially in the context of the SARS-CoV-2 pandemic blockade [5], with UPF accounting for more than half of total energy intake [6]. Consumption of UPF was significantly associated with a 23%, 25%, and 10% increased risk of colorectal, colon, and breast cancer, respectively; however, for rectal and prostate cancer, no significance was found [7]. Similarly, the consumption of more than five servings per week of fast food such as French fries or corn chips has been associated with an increased risk of developing colorectal cancer by more than four and three times, respectively [8]. Although there is knowledge about the relationship of UPF with cancer, the existing systematic reviews evaluating this association were limited by the small number of studies available [9, 10]; in addition to this, they only tend to focus on the most common types of cancer (breast, colorectal, and prostate, among others), ignoring the other types of cancers. This highlights the need for more studies on the impact of UPF on cancer development, as well as the need for an overview of the current state of the literature on the subject. In this sense, bibliometric studies can be of great help in achieving this objective since they are studies that allow a quantitative and qualitative analysis of a given topic, entity, or area of study and allow an evaluation of the predominant trends in that topic [11].

The results of this study will allow us to evaluate the recent panorama on the impact of UPF on the development of cancer and will not only serve as a basis for future studies on the subject but also for medical personnel to improve their care of patients by strengthening prevention and recommendations on diet and the impact it can have on their health. Previous bibliometric studies that have evaluated the topic of UPF have only reported its association with gut microbiota [12], obesity [13], or existing dietary measures [14].

To close these knowledge gaps, the aim of this study was to evaluate recent scientific productivity on the effect of UPF on cancer development.

## 2. Materials and Methods

### 2.1. Type of Study

A cross-sectional study was conducted with a scientometric mapping that explored the scientific literature between UPF and cancer.

### 2.2. Selection Criteria

For the study conducted, the following inclusion and exclusion criteria were established: documents published between 2018 and 2023, indexed in the Scopus database, that contained terms related to UPFs and cancer in their title or abstract, and that were available in their entirety for analysis were included. Papers that were not in English or Spanish due to language limitations; those that did not provide empirical data such as editorials, letters to the editor, commentaries, and opinions; those that did not focus specifically on the relationship between UPFs and cancer; and those that were not fully accessible were excluded. These criteria ensured that the papers selected for analysis were relevant and provided a complete and accurate overview of the scientific literature in the field of UPFs and cancer.

### 2.3. Data Collection

The search and review of the metadata was performed independently by two researchers to ensure accuracy and completeness. No duplicates were identified in the study since only one database, Scopus, which has robust mechanisms to avoid duplication of records, was analyzed. Regarding the quality of the articles included, especially the conference papers and notes, it is important to mention that since this is a scientometric study, the risk of bias was not assessed, since this assessment corresponds to systematic reviews or meta-analyses. Instead, it focused on the analysis of patterns and trends in the scientific literature on UPFs and cancer.

Data collection was conducted through a search of the Scopus database on February 4, 2024. A search strategy based on title and abstract terms is as follows: (“ultra-processed food∗” OR “processed food” OR “highly processed food” OR “industrially processed food” OR “fast food” OR “packaged food” OR “pre packaged food” OR “prepared food” OR “manufactured food” OR “commercially processed food” OR “sausages” OR “processed meats” OR “cured meats” OR “processed sausages” OR “packaged meats” OR “industrialized food” OR “preserved meats” OR “Foods, Processed” OR “Ultra-Processed Foods” OR “Ultra Processed Foods” OR “Processed Foods” OR “Food, Ultra-Processed” OR “Food, Ultra Processed” OR “Foods, Ultra-Processed” OR “Ultra-Processed Food”) AND (“cancer” OR “Tumor” OR “Neoplasm” OR “Tumors” OR “Neoplasia” OR “Neoplasias” OR “Cancer” OR “Cancers” OR “Malignant Neoplasm” OR “Malignancy” OR “Malignancies” OR “Malignant Neoplasms” OR “Neoplasm, Malignant” OR “Neoplasms, Malignant” OR “Benign Neoplasms” OR “Benign Neoplasm” OR “Neoplasms, Benign” OR “Neoplasm, Benign”) AND PUBYEAR >2017 AND PUBYEAR <2024.

### 2.4. Scientometric Analysis

A total of 662 articles, 189 reviews, 68 book chapters, 13 conference papers, and 9 notes were found. The papers were analyzed using SciVal and Bibliometrix in RStudio. In this study, several analyses were performed to gain a deeper understanding of the scientific literature in the field of UPFs and cancer. These analyses included the evaluation of scholarly output by country/region, the number of scholarly papers produced (scholarly output), the number of views (view count), the field-weighted citation impact (FWCI), and the total citation count (citation count). These indicators provide a comprehensive view of the influence and impact of research in the field.

## 3. Results

In the analysis conducted for the period 2018–2023, a comprehensive dataset that included 508 sources, such as journals, books, and other academic papers, was examined, resulting in a total of 662 papers. The annual growth rate during this period was calculated at 5.96%, indicating a sustained expansion of the literature in the specified time frame. The average age of the papers was 3.33 years, suggesting a relatively recent body of scholarly contributions. The average number of citations per paper was recorded at 18.56, underscoring the impact and relevance of the publications within the selected dataset. A total of 5524 authors contributed to the corpus, with 66 single-authored papers identified. Collaboration among authors was frequent, with an average of 7.78 coauthors per paper. International collaborations accounted for 27.23% of the collaborative efforts, highlighting the global dimension of the research networks. In terms of document types, the majority were articles (662), followed by book chapters (68), reviews (189), and various other formats (Table 1).

Author Inge Huybrechts, affiliated with the International Agency for Research on Cancer in France, was found to lead with 28 scholarly contributions, a citation per publication rate of 14, a field-weighted impact index of 4.28, and an *h*-index of 67. On the other hand, Marc J.R. Gunter, also from the same institution, presents 27 contributions, with an impressive citation rate per publication of 21.1 and an *h*-index of 73. Finally, Edward Luciano Giovannucci from Harvard University stands out with 16 contributions and a high citation rate per publication of 28.1, reflecting his influence in the field. Matthias Bernd Schulze of the University of Potsdam in Germany, Giovanna Masala of the Institute for the Study and Prevention of Cancer in Italy, and Mingyang Song of Harvard University, among others, also contribute significantly to research in this field, each contributing to the understanding of the relationship between UPF and cancer (Table 2).

In assessing impact and visibility, Harvard University in the United States leads with 52 contributions, a field-weighted impact of 3.39, and a remarkable number of views and citations, reflecting its global influence. The International Agency for Research on Cancer, a government agency in France, follows closely with 48 contributions, a field-weighted impact of 3.88, and a considerable number of views and citations, evidencing its relevance in cancer epidemiological research. In Spain, the Centro de Investigación Biomédica en Red and the Instituto de Salud Carlos III present similar results with 47 contributions each, highlighting their active participation in research on this topic. In addition, the Institut national de la santé et de la recherche médicale in France, the Institute Catala Oncologia in Spain, and the German Cancer Research Center in Germany contribute significantly to the field. These institutions demonstrate their commitment to research on UPFs, sausages, and cancer, both in terms of quantity and impact (Table 3).

**Figure 1:**
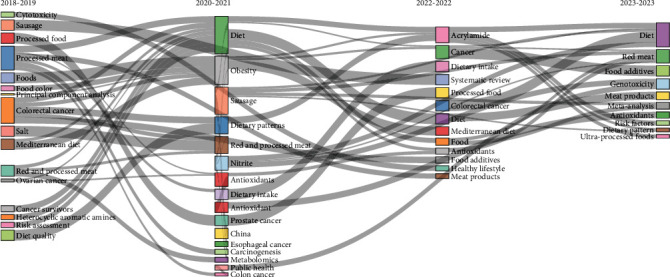
Thematic evolution on UPF and cancer.

Analysis of the thematic evolution revealed several changing dynamics; in 2018–2019, the focus shifted to topics such as cancer survivorship and colorectal cancer, exploring the relationship with diet and dietary patterns. During the 2020–2021 period, attention shifted to specific aspects of diet, including dietary quality, obesity, and processed meat. In addition, a focus on public health, food processing, and risk assessment was observed, evidencing an expansion of the subject towards more detailed and multidisciplinary aspects. In the last period, 2022–2023, the research diversified further, addressing areas such as the impact of UPFs, epidemiology, and cancer prevention recommendations, highlighting the continued evolution and breadth in the exploration of this complex relationship (Figure 1).

**Figure 2:**
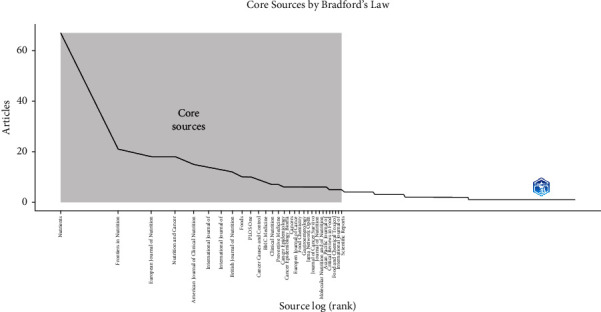
Core sources on UPF and cancer.

The journal “Nutrients” was found to occupy first place with a frequency of 67 in Zone 1, followed by “Frontiers in Nutrition” in second place with 21 and “European Journal of Nutrition” in third place with 18. In the same zone, “Nutrition and Cancer” and “American Journal of Clinical Nutrition” were ranked fourth and fifth, respectively, with a frequency of 18 and 15. In addition, it was noted that Zone 2 had its own set of prominent journals, such as “Advances in Nutrition,” “Antioxidants,” and “BMC Cancer,” all with frequencies of 4 (Figure 2).

**Figure 3:**
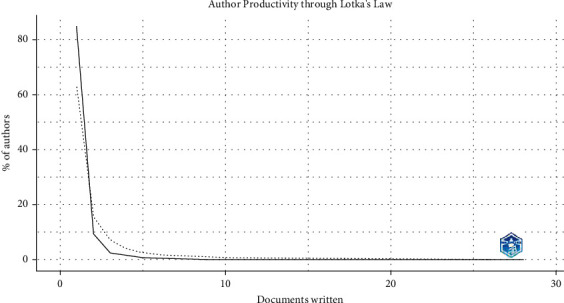
Author productivity on UPF and cancer.

The main findings revealed an asymmetric distribution in scientific production, supporting the validity of Lotka's law. It was observed that most of the papers were written by a single author, highlighting the prevalence of individual contribution in scientific production. As the number of authors increases, the frequency of papers decreases significantly, following a decreasing trend. This pattern evidenced the concentration of scientific activity in a select group of researchers, while more extensive collaborations are less frequent. These results underscore the importance of understanding the dynamics of authorship in scientific research and support the usefulness of Lotka's law to characterize the distribution of productivity among researchers (Figure 3).

**Figure 4:**
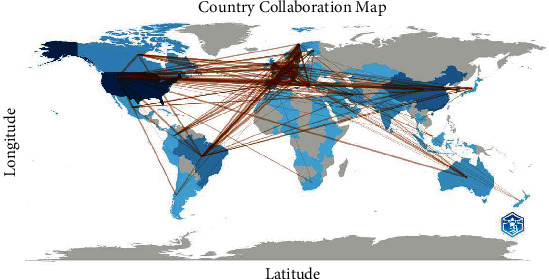
Country collaboration map on UPF and cancer.

The map of collaboration between countries revealed several significant findings in international scientific relations. First, the intense collaboration between Brazil and Canada stood out, with a remarkable frequency of seven collaborations. Likewise, strong connections were observed between Spain and multiple countries, with outstanding collaborations with France (24) and Germany (19). Scientific collaboration also extended across continents, evidenced by cooperation between Australia and Bangladesh (two) and China and Italy (three). Overall, these results reflect a global network of scientific collaboration, highlighting the diversity and breadth of connections between different nations in the field of research (Figure 4).

## 4. Discussion

In the present study, a scientometric mapping of the scientific literature between UPFs and cancer was performed, covering the period from 2018 to 2023. A sustained growth in literature production in this field was observed, with an annual growth rate of 5.96%. International collaboration was prominent, highlighting the contribution of countries such as Spain, France, Germany, Canada, and Brazil. The most prominent topics over the years were diet, cancer, and obesity. This analysis provides a comprehensive overview of the current state of research in this field, highlighting significant contributions from key authors and institutions and revealing the changing dynamics in research topics.

Mathilde Touvier, a French author affiliated with the French National Research Institute for Agriculture, Food and Environment (INRAE), was the author with the highest number of citations and the highest impact with an FWCI of 5.51 [15]. The most cited publication presented by this author on this study is the cohort conducted by Fiolet et al. [16], where the association between the consumption of UPFs and the risk of cancer in 2228 patients was evaluated.

The French governmental institution International Agency for Research on Cancer was the institution with the highest impact (FWCI: 3.88) on the topic of UPF and cancer. This institution, located in Lyon (France), is part of the World Health Organization (WHO) and presents several contributions to cancer research, such as the creation of the Global Cancer Observatory (GCO) that allows epidemiological analysis of 185 countries and 36 types of cancers by age and sex [1]. In addition to this, this institution promotes consensus among the various representatives of the main institutions involved in the classification of tumors and related fields through the International Collaboration for Cancer Classification and Research (IC3R) to identify and discuss multinational challenges in comparability and establishment of data and standards, quality management, and evidence assessment, as well as to develop collaborative plans to address these challenges [17]. Similarly, this institution writes annual monographs to update evidence syntheses on cancer research and promote transparency in cancer hazard identification [18].

The journal Nutrients had the highest productivity, followed by the journal Frontiers in Nutrition. Among the most cited publications in Nutrients, and directly related to the topic of this study, was a narrative review [19] with 332 citations that evaluated the effect of UPF on general health, where cancer was highlighted as a possible effect of poor diet. It is worth mentioning that this journal has also been reported as one of the most productive and popular on general nutrition topics in the current medical literature [20], so it would not be surprising if it is also one of the most productive on UPF on cancer issues.

Even though research seems to be concentrated in only a few research groups, collaboration between countries was reported to be more oriented towards an international panorama. In general, a large collaboration network was reported between European countries (mainly Spain, France, and Germany) and American countries such as the United States; it should also be added that these same countries are the ones that presented the highest productivity about this study. This may be due to these countries' own policies about UPF. In most European countries, a decreasing trend of up to 15% in UPF consumption has been reported in recent years [21], which represents an adequate execution of diet and education programs in the population, which, to be implemented, require an adequate investigation of the population's situation. On the contrary, in the United States, the issue of UPF consumption is a fairly recent topic in their health policies since most of them only consider it within unhealthy foods and it is not evaluated independently [22]; the fact that they present a good collaboration network would strengthen the study of the population outlook in that country and in the long term would allow the improvement of their public policies.

Regarding the thematic evolution, while in the beginning, the research seemed to focus on the effect such as cancer survival or colorectal cancer; in later years, there was a focus on the cause such as public health, food processing, and risk assessment, and finally, in the last years, it focused on the direct impact of UPFs, their epidemiology, and cancer prevention. The fact that in previous years, one of the main topics was colorectal cancer is in line with the literature, which reports that although poor diet has been associated with the development of cancers, this research tends to focus on the most frequent types [23], minimizing the other types. Furthermore, the change that has occurred in research so that between 2022 and 2023 it will focus on a direct study of UPF can be explained by the evolution of food in the world; at first, food was based only on minimally processed foods, but now, due to the great industrial activity and globalization, the consumption of foods high in fat and with greater processing has been prioritized due to their greater profitability and lower cost in the long term [24].

This study had some limitations. First, the search was limited only to the Scopus database and between 2018 and 2023, which may have excluded relevant articles from other databases, gray literature, or publications prior to that date. In addition, the classification of the articles into articles, review, and letter does not reflect the methodology of the articles. However, this study also presents several strengths. First, Scopus is a multidisciplinary database that not only covers many high-quality journals but, compared to other databases, provides significant results with the search strategy [25, 26], thus ensuring high quality and specificity in the publications evaluated in this study. Secondly, classifying the studies according to the type of publication allows a more complete view of the subject field evaluated. Finally, this is the first study that evaluates productivity on UPF consumption and its effect on cancer development, so it is expected that our results will provide a crucial overview on knowledge gaps and serve as a basis for the development of future studies and better public policies focused on prevention and education on the importance of diet in health.

## 5. Conclusion

Within the limitations of this study, it is concluded that during the period analyzed (2018–2023), the average age of the articles, 3.33 years, suggests a relatively recent scholarly contribution. The considerable mean of 18.56 citations per article underscores the impact and relevance of the publications in the selected dataset. International collaboration, representing 27.23% of the collaborative effort, highlights the global dimension of the research networks. Most influential authors, Inge Huybrechts, Marc J.R. Gunter, and Edward Luciano Giovannucci, lead with significant contributions, reflecting their influence in the field. The institutions, led by Harvard University, demonstrated their leadership in both quantity and impact. Research has expanded into multidisciplinary aspects, such as microbiota, antioxidants, and the NOVA classification of UPFs. At the international level, scientific collaborations between countries reveal a global research network, highlighting the intense collaboration between Brazil and Canada.

## Figures and Tables

**Table 1:** Main information of scientific literature.

**Description**	**Results**
Period	2018–2023
Sources (journals, books, etc.)	508
Annual growth rate (%)	5.96
Document average age	3.33
Average citations per doc	18.56
References	51,990
Keyword plus	5433
Author's keywords	2205
Authors	5524
Authors of single-authored docs	66
Single-authored docs	67
Coauthors per doc	7.78
International coauthorships (%)	27.23
Article	662
Book	1
Book chapter	68
Conference paper	13
Conference review	1
Editorial	3
Erratum	3
Letter	5
Note	9
Review	189
Short survey	1

**Table 2:** Top 10 most representative authors on UPF and cancer.

**Author**	**Affiliation**	**Region**	**Scholarly output**	**Citations per publication**	**Field-weighted citation impact**	**h** **-index**
Huybrechts, Inge	International Agency for Research on Cancer	France	28	14	4.28	67
Gunter, Marc J.R.	International Agency for Research on Cancer	France	27	21.1	4.45	73
Tjønneland, A. Marie	Danish Cancer Society	Denmark	18	19.9	3.81	142
Tumino, Rosario	Hyblean Association for Epidemiological Research	Italy	18	17.9	3.97	133
Weiderpass, Elisabete	International Agency for Research on Cancer	France	18	21.3	4.18	128
Giovannucci, Edward Luciano	Harvard University	United States	16	28.1	4.21	192
Schulze, Matthias Bernd	University of Potsdam	Germany	15	14.3	3.57	91
Masala, Giovanna	Institute for the Study and Prevention of Cancer	Italy	14	15.6	2.62	77
Song, Mingyang	Harvard University	United States	14	55.7	5.28	57
Touvier, Mathilde	INRAE	France	14	73.5	5.51	77

**Table 3:** Top 10 most representative institutions on UPF and cancer.

**Institution**	**Sector**	**Country/region**	**Scholarly output**	**View count**	**Field-weighted citation impact**	**Citation count**
Harvard University	Academic	United States	52	2028	3.39	2007
International Agency for Research on Cancer	Government	France	48	2270	3.88	1856
Centro de Investigación Biomédica en Red	Academic	Spain	47	2167	2.44	1082
Instituto de Salud Carlos III	Academic	Spain	47	2167	2.44	1082
Institut national de la santé et de la recherche médicale	Government	France	34	1624	3.48	1437
Institute Catala Oncologia	Government	Spain	31	1672	2.86	748
German Cancer Research Center	Government	Germany	28	1435	2.96	447
INRAE	Government	France	28	1644	3.17	1206
IRCCS Fondazione Istituto Nazionale per lo studio e la cura dei tumori–Milano	Medical	Italy	28	1376	2.67	509
German Institute of Human Nutrition Potsdam-Rehbruecke	Government	Germany	27	1571	3.43	704

## Data Availability

The data are available upon request to the corresponding author.
